# Assessment of radio(chemo)therapy-related dysphagia in head and neck cancer patients based on cough-related acoustic features: a prospective phase II national clinical trial (ACCOUGH-P/A trial)

**DOI:** 10.1186/s13063-023-07660-y

**Published:** 2023-09-29

**Authors:** Sofiana Mootassim-Billah, Gwen Van Nuffelen, Jean Schoentgen, Marc De Bodt, Dirk Van Gestel

**Affiliations:** 1 Department of Radiation Oncology, Speech Therapy, Institut Jules Bordet, Hôpital Universitaire de Bruxelles, Université Libre de Bruxelles, Brussels, Belgium; 2 Department of Otolaryngology and Head and Neck Surgery, University Rehabilitation Center for Communication Disorders, Antwerp University Hospital, Antwerp, Belgium; 3 Department of Translational Neurosciences, Faculty of Medicine and Health Sciences, University of Antwerp, Antwerp, Belgium; 4 Department of Logopaedics and Audiological Sciences, Faculty of Medicine and Health Sciences, University of Ghent, Ghent, Belgium; 5 Department of Biomechatronics, Université Libre de Bruxelles, Brussels, Belgium; 6 Department of Radiation Oncology, Head and Neck Unit, Institut Jules Bordet, Hôpital Universitaire de Bruxelles, Université Libre de Bruxelles, Brussels, Belgium

**Keywords:** Head and neck cancer patients, Radiation-associated dysphagia, (Chemo)radiotherapy, Cough, Assessment, Acoustic analysis

## Abstract

**Background:**

Radiation-associated dysphagia is defined as impaired swallowing efficiency/safety following (chemo)radiotherapy in head and neck cancer patients. In a dysphagia framework, impaired coughing may lead to lung aspiration and fatal lung infection. Although cough efficacy is a predictor of the risk of aspiration, cough investigation is minimal in patients with radiation-associated dysphagia. Because cough is a transient signal, existing software for speech analysis are not appropriate. The goal of our project is to develop an assessment method using acoustic features related to voluntary and reflexive coughs as biomarkers of the risk of penetration/aspiration in patients with radiation-associated dysphagia.

**Methods:**

Healthy subjects and head and neck cancer patients with and without dysphagia will produce voluntary coughs, throat clearings and reflexive coughs. Recordings will be made using an acoustic microphone and a throat microphone. The recorded signals will be manually segmented and subsequently analysed with a software under development. Automatic final segmentation enables to measure cough duration. The first method of analysis includes temporal features: the amplitude contour, the sample entropy and the kurtosis. These features report respectively the strength, the unpredictability (turbulence noise due to the air jet) and the impulsive quality (burst) of the signal. The second method of analysis consists of a spectral decomposition of the relative cough signal energy into several frequency bands (0–400 Hz, 400–800 Hz, 800–1600 Hz, 1600–3200 Hz, > 3200 Hz). The primary outcome of this exploratory research project is the identification of a set of descriptive acoustic cough features in healthy subjects as reference data (ACCOUGH). The secondary outcome of this research in head and neck cancer patients with radiation-associated dysphagia includes the identification of (1) a set of descriptive acoustic cough features as biomarkers of penetration-aspiration (ACCOUGH-P/A), (2) swallowing scores, (3) voice features and (4) aerodynamic cough features.

**Discussion:**

This study is expected to develop methods of acoustic cough analysis to enhance the assessment of radiation-associated dysphagia in head and neck cancer patients following (chemo)radiation.

**Trial registration:**

International Standard Randomized Controlled Trials Number (ISRCTN) registry ISRCTN16540497. Accepted on 23 June 2023.

## Administrative information

Note: The numbers in curly brackets in this protocol refer to the SPIRIT checklist item numbers. The order of the items has been modified to group similar items (see http://www.equator-network.org/reporting-guidelines/spirit-2013-statement-defining-standard-protocol-items-for-clinical-trials/).

**Table:** 

Title {1}	Assessment of radio(chemo)therapy-related dysphagia in head and neck cancer patients based on cough-related acoustic features: a prospective phase II national clinical trial (ACCOUGH-P/A trial)
Trial registration {2a and 2b}	ISRCTN ID 16540497. Registration was done retrospectively and accepted on 23 June 2023.
Protocol version {3}	V.04 4 March 2020
Funding {4}	This study is funded by the Department of Radiation-Oncology at the Jules Bordet Institute and received financial support from L’Association Jules Bordet.
Author details {5a}	Sofiana Mootassim-Billah; MSc (corresponding author) Department of Radiation Oncology, Speech Therapy, Institut Jules Bordet, Hôpital Universitaire de Bruxelles, Université Libre de Bruxelles, Brussels, Belgium Mail: s.mootassim-billah@hubruxelles.be Tel: + 32.2.541.72.02 Gwen Van Nuffelen; PhD Department of Otolaryngology and Head and Neck Surgery, University Rehabilitation Center for Communication Disorders, Antwerp University Hospital, Antwerp, Belgium Department of Translational Neurosciences, Faculty of Medicine and Health Sciences, University of Antwerp, Antwerp, Belgium Department of Logopaedics and Audiological Sciences, Faculty of Medicine and Health Sciences, University of Ghent, Ghent, Belgium Mail: gwen.vannuffelen@uza.be Jean Schoentgen; PhD Department of Biomechatronics; Université Libre de Bruxelles, Brussels, Belgium Mail: jean.schoentgen@ulb.be Marc De Bodt; PhD Department of Otolaryngology and Head and Neck Surgery, University Rehabilitation Center for Communication Disorders, Antwerp University Hospital, Antwerp, Belgium Department of Translational Neurosciences, Faculty of Medicine and Health Sciences, University of Antwerp, Antwerp, Belgium Department of Logopaedics and Audiological Sciences, Faculty of Medicine and Health Sciences, University of Ghent, Ghent, Belgium Mail: marc.de.bodt@infonie.be Dirk Van Gestel; MD, PhD Department of Radiation Oncology, Head and Neck Unit, Institut Jules Bordet, Hôpital Universitaire de Bruxelles, Université Libre de Bruxelles, Brussels, Belgium Mail: dirk.vangestel@hubruxelles.be
Name and contact information for the trial sponsor {5b}	The Jules Bordet Institute and the Antwerp University Hospital.
Role of sponsor {5c}	The supporting sources of funding had no role in the design, execution, analysis and interpretation of the data.

## Introduction

### Background and rationale {6a}

Radiation-associated dysphagia (RAD) is impaired swallowing safety/efficiency following (chemo)radiotherapy [1–3]. RAD has been reported by up to 79% of head and neck cancer (HNC) patients and may persist for more than 10 years after (chemo)radiotherapy ((C)RT) [4–6]. The two clinical hallmarks of RAD are residue (food sticking in the oral cavity, pharynx or larynx) and penetration/aspiration (P/A)—an entry of material in the airways [7, 8]. P/A results ideally in a cough reflex that serves an important protective role for the airways and lungs by expelling materials.

Cough is a sensorimotor behaviour defined as a deep inspiration followed by closure of the glottis, forced expiratory effort and then opening of the glottis with expiration [9]. Neurologically, voluntary coughs and throat clearings produced upon request are initiated in the cerebral cortex. A voluntary throat clearing—a manoeuver that clears the pharynx and larynx from pooling (pre-swallow secretions and post-swallow food residue)—differs from a voluntary cough as it is produced without any prior inspiration and requires partial vocal fold closure only [10, 11]. Pooling is regarded as a clinical predictor of prandial P/A in the lower respiratory tract [12–14], so efficient coughing, throat clearing and dry swallowing are considered as crucial spontaneous or voluntary defensive strategies to clear the airway [13]. A reflexive cough is stimulated by a biological need to protect the airways and is triggered via direct activation of receptors on airway sensory nerves (vagus nerves) by mechanical and/or chemical stimuli [11, 15, 16] to the brainstem, bypassing cortical control completely [17]. From the brainstem, efferent nerves (vagus, phrenic and spinal motor nerves) are stimulated and activate thoracic and abdominal musculature and laryngeal structures responsible for a reflexive cough [17].

In HNC patients with RAD, the elicited cough is often ineffective, intermittently ineffective or absent following (C)RT [18]. Cough ineffectiveness is due to a combination of factors such as sensory deterioration of the laryngopharyngeal structures and cough strength impairment caused by weakness of the pharyngeal muscles, muscle atrophy, fibrosis and low levels of substance P—a neuropeptide present in human airway nerves that mediates the cough response [3, 18, 19]. Silent aspirations—no cough reflex is triggered despite entry in the trachea—and silent penetrations—no cough reflex is triggered despite entry in the larynx—are respectively observed in up to 83% of aspiration and 100% of penetration occurrences in HNC patients with RAD [20–23]. Consequently, aspiration pneumonia is the most severe complication of cough ineffectiveness because it comes with a mortality rate of up to 34.6% [22–24]. Given these vital issues, cough efficacy is considered as a major predictor of aspiration risk in HNC patients with RAD [18, 22–26].

In daily clinical practice, the established cough assessment is based on a perceptual auditory assessment of voluntary coughs, voluntary throat clearings and/or reflexive coughs [27]. Recently, voluntary cough has been found to be a low predictor of aspiration risk [28–30]. Besides, a study by Laciuga et al. have demonstrated that the subjective nature of perceptual cough evaluation results in many inconsistencies with regard to cough scoring, particularly for rating cough strength or effectiveness and distinguishing cough from throat clearing [10]. Inherent to the nature of reflexive coughs, they only occur in case of non-silent P/A. Subsequently, natural reflexive coughs cannot be judged in each clinical swallowing evaluation, adding another weakness to the perceptual evaluation of coughs in the framework of RAD and aspiration. For this reason, the implementation of a systematic assessment of induced reflexive cough during a clinical swallowing evaluation might enhance the detection of ineffective coughing in this population with frequent silent P/A. Indeed, an inhalation cough challenge is reported to enable the examination of the cough reflex threshold, the laryngopharyngeal sensitivity and the cough effectiveness to protect the airway in different populations [29, 31–34]. Besides, a standardized inhalation cough challenge facilitates universal interpretation and comparison of data [31].

In research, cough is objectively measured by collecting airflow measures using a facemask or pipe coupled to a filter and connected to a digital spirometer [29, 35, 36]. The peak expiratory flow rate (PEFR) in litres/second (l/s) and the total expired volume (TEV) in litres (l) have been reported to be the most reliable predictors of P/A risk obtained in the framework of an aerodynamic cough assessment [29, 33, 37]. Although this method is considered to be the gold standard for reliably assessing coughing, an aerodynamic equipment is not widely available amongst clinicians, its set-up is time-consuming and it is impractical for routine clinical assessment, i.e. evaluation of these biomarkers during swallowing or food intake.

To our knowledge, no objective acoustic features of coughing and throat clearing have been reported to be relevant to dysphagia. Novel methods enabling the detection of acoustic cough features that could be suitable for clinical assessment in a natural setting (e.g. bedside) are thought to add value to daily clinical practice regarding patients with RAD.

Another frequent aspect of the clinical diagnostic examination of swallowing is the perceptual assessment of voice quality immediately following deglutition [38]. Changes in voice quality are assumed to inform on the possible accumulation of saliva or food at the level of the vocal folds [38–40]. Waito et al. report that a normophonic voice after swallowing reflects a lack of P/A [38]. However, research shows that the relation between swallowing impairments and changes in perceived voice quality (e.g. wet voice) is unclear [38, 41]. Besides, a systematic review reported that it is not possible to obtain a consensus regarding the use of vocal quality as an indicator of swallowing impairment [42]. Indeed, standardized methods and consensual protocols regarding voice investigation in the framework of dysphagia are lacking because no acoustic voice features of reasonable evidence of dysphagia have been found, although some studies have reported that an aspiration risk may be correlated with acoustic features such as vocal jitter (relative average perturbation of the glottal cycle lengths) and harmonics-to-noise ratio [40, 43].

The exploration of specific acoustic voice features as predictors of the P/A risk in HNC patients with RAD could be a reliable and easily implementable complementary method of the cough assessment during a clinical swallowing evaluation. The combination of acoustic voice and cough features is expected to enhance the accuracy of P/A prediction. Indeed, some studies have reported that considering the cough assessment individually reduces the reliability of P/A prediction [28, 44].

### Objectives {7}

The primary objective of this study is to develop an ecological and non-invasive assessment method for dysphagia and P/A in HNC patients using acoustic features related to voluntary and reflexive coughs as biomarkers of dysphagia and penetration/aspiration in this population.

Secondary objectives involve the investigation of the relation between acoustic and aerodynamic cough features as well as between acoustic cough and voice features, extending our insight into the pathophysiology of dysphagia in this population.

### Endpoints of this study

1. Construction of a set of descriptive acoustic cough features (ACCOUGH)
  - Identification of a new set of acoustic cough features in a sample of a healthy population (reference data)
  - Comparison between acoustic voluntary and reflexive cough features as well as acoustic features of throat clearing
  - Selection of descriptive acoustic cough features
2. Validation of the ACCOUGH features as biomarkers of P/A in HNC patients (ACCOUGH-P/A)
  - Identification of the ACCOUGH features in samples of HNC patients
  - Investigation of the correlation between ACCOUGH features and observed P/A in HNC patients
3. Correlation between ACCOUGH and aerodynamic cough features
  - Investigation of the correlation in a sample of a healthy population
  - Investigation of the correlation in samples of HNC patients
4. Investigation of the correlation between ACCOUGH-P/A and acoustic voice features in samples of HNC patients
5. Investigation of the relation between voice quality abnormalities and observed P/A in samples of HNC patients

### Trial design {8}

This exploratory study is multicentre and will be conducted at the Jules Bordet Institute and the Antwerp University Hospital, both located in Belgium. All measurements will be carried out once, in a single session per subject. HNC patients will be eligible for 3 months post-oncological treatment.

## Methods: participants, interventions and outcomes

### Study setting {9}

In total, 90 subjects will participate in this study: 40 healthy participants, 40 HNC patients with RAD and a pilot group of 10 HNC patients with no reported or diagnosed RAD. We plan to include a pilot group of HNC patients without RAD because we would like to examine if acoustic cough features as biomarkers of dysphagia are different from acoustic cough features in HNC patients without dysphagia following the same oncological treatment.

### Eligibility criteria {10}

Healthy subjects will be recruited on a voluntary basis from a pool of healthy volunteers who will be made aware of the study (hospital staff and extra hospital parties). HNC patients will be recruited by a radiation oncologist and a speech-language pathologist (SLP). The SLP will explain the study protocol to the participants. All participants must provide a written, informed consent before any study procedures occur. The SLP will carry out all the cough and voice recordings. A fiberoptic endoscopic evaluation of swallowing (FEES) will be performed by an experienced head and neck surgeon.

Below are the inclusion and exclusion criteria:

The following are the inclusion criteria for healthy participants:

- Aged 18 years minimum
- Lack of relevant medical complaints
- Lack of cognitive complaints

The following are the exclusion criteria for healthy participants:

- History of head and neck cancer
- Dysphagia according to the Yale Swallow Protocol [45]
- Dysphonia (G > 0 on GRBAS-I scale [46])
- History of smoking within less than one year of the data recording
- Acute or chronic respiratory disease (e.g. chronic obstructive pulmonary disease or asthma)

The following are the inclusion criteria for HNC patients:

- Aged 18 years minimum.
- Lack of cognitive complaints.
- Eligible tumour sites: oral cavity, oropharynx, hypopharynx, nasopharynx and larynx. All tumour sites will be diagnosed by a team of experienced head and neck surgeons, head and neck radiation oncologists, oncologists, radiologists and anatomopathologists based on clinical examination, dual-energy CT scan and PET-CT scan.
- Treated with (chemo)radiotherapy. All patients will be treated with 32 fractions (5 fractions per week) of slightly accelerated simultaneous integrated boost (SIB) intensity-modulated RT (IMRT). Patients who will be concomitantly treated with chemotherapy will receive either cisplatin 100 mg/m^2^ IV (days 1 and 22) or weekly cisplatin 40 mg/m^2^ IV (days 1, 8, 15, 22, 29, and 36). Patients treated for nasopharyngeal cancer may sometimes receive induction chemotherapy (cisplatin + gemcitabine) before the start of concurrent IMRT and weekly cisplatin 40 mg/m^2^ IV (6 cycles).
- Main group (*N* = 40): diagnosed with radiation-associated dysphagia at least 3 months post-oncological treatment, based on the Common Terminology Criteria for Adverse Events (CTCAE score > 0) [47].
- Pilot group (*N* = 10): not diagnosed with radiation-associated dysphagia at least 3 months post-oncological treatment, based on CTCAE (score 0) [47].
- In clinical remission: total disappearance of the tumour (total response to treatment) based on clinical examination, dual-energy CT scan and PET-CT scan.

The following are the exclusion criteria for HNC patients:

- Dysphagia prior to oncological treatment
- Acute or chronic respiratory disease (e.g. chronic obstructive pulmonary disease or asthma)
- History of major surgery in the head and neck region
- Recurrent carcinoma in the head and neck region

### Who will take informed consent? {26a}

All participants must provide written informed consent before being part of any study procedure. When obtaining and documenting informed consent, the investigator must comply with the applicable regulatory requirement(s) and must adhere to Good Clinical Practice (GCP) and to the ethical principles laid down in the Declaration of Helsinki. Prior to the beginning of the trial, the investigator must have the Jules Bordet Institute and Antwerp Hospital Ethics Committees’ written approval/favourable opinion of the written informed consent form and of any other written information to be provided to subjects.

Any revised written informed consent form and written information must receive in advance the Ethics Committee’s approval/favourable opinion.

The language used in the oral and written information about the trial, including the written informed consent form, will be as non-technical as practical and should be understandable to the subject or the subject’s legal representative and the impartial witness, when applicable. The trial details will be explained orally in a quiet consulting room.

Before informed consent may be obtained, the investigator, or a person designated by the investigator, must give the subject 1 week (7 days) to inquire about the details of the trial and to decide whether or not to participate in the trial. All questions about the trial must be answered to the satisfaction of the subject or the subject’s legal representative.

Prior to a subject’s participation in the trial, the written informed consent form must be signed and dated by the subject or by the subject’s legal representative and by the person who conducted the informed consent discussion. A copy of the signed informed consent form will be given to each participant.

### Additional consent provisions for collection and use of participant data and biological specimens {26b}

Not applicable. Participant data and biological specimens are not planned to be used in future studies.

### Interventions

#### Explanation for the choice of comparators {6b}

This study will compare HNC patients (*N* = 40) to healthy subjects as a control group (*N* = 40). Indeed, this study is exploratory, and reference data are therefore needed. Healthy participants will be paired with HNC patients according to the Clinical Frailty Scale (CFS), a clinical judgement-based frailty tool [48, 49]. This scale includes the evaluation of comorbidity, function and cognition to calculate a frailty score ranging from 1 (very fit) to 9 (terminally ill). HNC patients with RAD (*N* = 40) will also be compared to a pilot group of HNC patients without RAD (*N* = 10).

#### Intervention description {11a}

All healthy participants and HNC patients will carry out the same tasks, except that HNC patients will also undergo a fiberoptic endoscopic evaluation of swallowing (FEES), simultaneously with the acoustic recording of natural reflexive coughs via a skin-contact microphone. The data collected are presented in Table 1.

**Table 1:** Data collected in the ACCOUGH-P/A trial

Time point	Enrolment	Participation in the study
	***Healthy subjects: no specific time point*** ***HNC patients: minimum 3 months post-oncological treatment***	***Healthy subjects: no specific time point*** ***HNC patients: one visit, minimum 3 months post-oncological treatment***
**Enrolment**		
*Eligibility screening*^a^* (4 weeks maximum before enrolment)*	X	
*Common terminology criteria for adverse events score*	X^b^	
*Informed consent*	X	
**Assessments (one assessment per participant)**		
*Reporting of sociodemographic data and medical history*		X
*Acoustic and aerodynamic recordings of voluntary coughs, voluntary throat clearings and induced reflexive coughs*		X
*Acoustic voice recordings of sustained vowels [a]*		X
*Fiberoptic evaluation of swallowing including:* - *Acoustic recordings of natural reflexive coughs* - *Swallowing scores* - *Pre/post-swallow acoustic voice recordings of sustained vowels [a]*		X^b^

^a^The screening will take approximately 2 h per participant (reading of the medical record and meeting with patients during follow-up oncological consultations)

^b^Assessment carried out only with head and neck cancer patients

Participants will be seated in an audiometric booth while producing other cough and voice samples.

##### Signal recordings

Cough and voice samples recorded from all participants will be as follows:

- Five voluntary single coughs
- Five voluntary single throat clearings
- A minimum of two induced reflexive coughs
- A sustained vowel [a] before and after swallowing a sip of water of 20 ml

Cough and voice samples recorded only from HNC patients during FEES are as follows:

- Natural reflexive coughs
- Sustained vowels [a] before and after each swallowing trial

##### Equipment

Acoustic and aerodynamic recordings will be carried out.

(A) Acoustic equipment: The acoustic recordings will be made simultaneously using a skin-contact microphone (Albrecht AE 38 S2) and a professional quality acoustic free-standing microphone (AKG Perception 420 Omnidirectional). The intensity in dB will be recorded by an external sound level metre (Bruel and Kjaer 2236). The free-standing microphone and the external sound level metre will be placed at 40 cm to the right of the mouth of the participant. Only the skin-contact microphone will be used to record induced reflexive coughs because of the presence of the anaesthesia facemask through which citric acid will be delivered (see more details in section (B) Aerodynamic equipment). Natural reflexive coughs and sustained vowels [a] produced by HNC patients during FEES will be recorded with the skin-contact microphone only to avoid the recording of environmental or equipment noise. Acoustic cough and voice signals will be recorded with an HP ProBook computer (Hewlett-Packard Company, USA) using the computer program PRAAT and the preamplifier 2-channel interface Presonus Audiobox USB 96 Audio, with a sampling frequency of 44.1 kHz.
(B) Aerodynamic equipment: The aerodynamic recordings will be carried out with an anaesthesia facemask connected to a spirometer Pocket-Spiro USB (Medical Electronic Construction, M.E.C, Belgium). Cough will be induced using a differential pressure transducer with a one-way inspiratory valve for a nebulizer connection. The nebulizer will deliver citric acid during a 2-s inspiration. Each participant will complete a maximum of 5 challenges of increasing concentrations of citric acid: saline, 30 mM or 5.8 mg/ml citric acid, 100 mM or 19.2 mg/ml citric acid, 300 mM or 58 mg/ml citric acid and 1000 mM or 192 mg/ml citric acid as described in Janssens et al. [50]. To avoid tachyphylaxis (a decreased response to repeated stimulation), concentrations of citric acid will be delivered incrementally, and all inter-trial intervals will last for a minimum of 60 s. Reflexive coughs induced in response to the challenges will be monitored to define the lowest concentration at which 2 or more successive coughs (C2 threshold) are triggered after one single inspiration [31]. Aerodynamic cough samples will be recorded with an HP ProBook computer (Hewlett-Packard Company, USA) using the computer program M.E.C. PDI (Medical Electronic Construction, M.E.C, Belgium).

##### Acoustic cough analyses

All recorded cough and throat-clearing signals will be analysed with a software developed for the purpose of this study and written in the Python programming language.

(A) Segmentation: Cough samples are segmented manually into single coughs leaving silent intervals before and after each signal. Subsequent automatic segmentation is performed via the signal contour by assigning to the onset the first contour sample and to the offset the last contour sample the value of which is larger than − 30 dB with regard to the signal contour maximum. Prior to analysis, the segmented cough signals are normalized so that the average energy of the signal equals 1.
(B) Spectral analysis: Cough signals are transient signals (average duration of 0.3 s). As a consequence, conventional spectrograms are an unsatisfactory representation. Spectrograms are suitable for locally stable sounds such as speech signals that comprise quasi-stable targets connected by transitions. Also, features available in standard analysis tools are suitable for sustained sounds only. The cough signal changes quickly and incessantly over a brief period of time. Given the transient nature of the cough signal, our software focus on a limited number of isolated frequency bands, whose spectral energies are reported band by band. By lowering the number of frequency bands (lower frequency resolution), we are able to examine the temporal evolution of the signal band by band (higher temporal resolution). A filter bank based on the discrete cosine transform (DCT) divides the signals into frequency bands, the boundaries of which are 0–400 Hz, 400–800 Hz, 800–1600 Hz and 1600–3200 Hz as well as the interval between 3200 Hz and half the sampling frequency (22,050 kHz). The DCT periodically extends the signal by pivoting it with respect to its onset and offset so that the extended signal is even. The difference between the discrete cosine transform and the discrete Fourier transform is that the former avoids spectral artefacts that would be caused by concatenating rapid high-amplitude onsets (bursts) with gradual low-amplitude offsets. The cough signal can be accurately decomposed using a DCT, meaning that the energy of the original cough signal and the sum of the energies of the band-filtered signals are identical [51]. The spectral features are the relative signal energies in the previously mentioned bands. The number of unidirectional zero-crossings is used to estimate the average frequency in each band. The relative band energies are used to weigh the individual band frequencies before being summed. The weighted frequency is a close approximation of the spectral centroid, which divides the signal spectrum into two equal-energy halves.
(C) Temporal analysis: The temporal analysis involves the evolution with time of the contours of the cough signal amplitude, the sample entropy and the kurtosis.

The *amplitude contour* reports the relative strength of the cough signal. Because of the normalization, the average amplitude is not relevant. Normalization removes the influence of the microphone position and pre-amplifier gain on the cough signal features. An independent sound level metre is used to report the intensity of the cough signal in decibels (dB).

The *contour of the sample entropy* reports the degree of unpredictability. The sample entropy enables segregating analysis frames according to whether they report turbulence noise or locally periodic oscillations because the former is expected to be less predictable than the latter [52].

The *kurtosis* can be interpreted in terms of the peakedness of the histogram of the sample values in an analysis frame. Sample histograms that are flatter than normal have kurtosis values between 3 and 0. Histograms that are peakier than normal have kurtosis values greater than 3. It is expected that onset bursts have greater kurtosis values than turbulence noise or oscillations [53].

The *shape of the contours* of the cough amplitude, sample entropy and kurtosis is described using the first three DCT coefficients. The pattern of the first three co-sinusoidal basis functions shows that the first coefficient is the contour average. The second coefficient describes the contour slope. A positive coefficient value indicates a signal contour that decreases with time. The third coefficient reports the contour curvature. A positive coefficient value indicates a downward-upward (convex) curvature of the contour, whereas a negative value indicates an upward-downward (concave) curvature of the contour, with respect to the horizontal.

##### Aerodynamic cough analyses

Cough samples will be analysed using the software PDI developed by M.E.C. Medical Electronic Construction (M.E.C, Belgium). For each cough sample, this software extracts automatically the peak expiratory flow rate (PEFR) in litres/second (l/s) and the total expired volume (TEV) in litres (l), which are reported to be the most reliable predictors of P/A risk obtained in the framework of an aerodynamic cough assessment [29, 33, 37].

##### Acoustic voice analyses

Sustained vowels [a] will be analysed using the software PRAAT, and the intensity will be recorded with an autonomous sound level metre (Bruel and Kjaer 2236). Extracted voice features will be the fundamental frequency (F0), harmonics-to-noise ratio (HNR), jitter, shimmer and intensity (dB).

#### Criteria for discontinuing or modifying allocated interventions {11b}

Participants will be discontinued from the study at any time if:

- They withdraw their consent to participate
- They lose their cognitive capacity to consent to trial participation
- Their medical condition is deteriorating

#### Strategies to improve adherence to interventions {11c}

Each recording session will be supervised, face-to-face, by the first author, a certified SLP. All signal recordings as well as FEES will be performed on the same day in a single session to motivate subjects to participate in this study.

#### Relevant concomitant care permitted or prohibited during the trial {11d}

At the time of assessment, head and neck cancer patients might receive concomitant care from a SLP for dysphagia rehabilitation because this study explores acoustic features as biomarkers of P/A, not the swallowing function improvement under one specific intervention.

#### Provisions for post-trial care {30}

Provisions for post-trial care are not planned because there is no adverse effect or harm expected with this trial participation.

### Outcomes {12}

#### Primary outcome measure

##### ACCOUGH

The primary outcome is the identification of a set of descriptive acoustic cough features in healthy subjects as reference data for comparison with HNC patients. This outcome is addressed via two methods of analysis: a temporal analysis and as spectral analysis. The temporal features include the signal duration as well as shape features of the amplitude, the sample entropy and the kurtosis contours. The spectral features are the relative signal energies in the bands (0–400 Hz, 400–800 Hz, 800–1600 Hz, 1600–3200 Hz), the interval between 3200 Hz and half the sampling frequency (22,050 kHz) and the weighted frequency.

#### Secondary outcome measures

The secondary outcome measures of this research include (1) ACCOUGH-P/A: acoustic cough features as biomarkers of P/A, (2) swallowing scores, (3) voice features and (4) aerodynamic cough features.

##### ACCOUGH-P/A

The ACCOUGH features will be identified in HNC patients, and the correlation between observed P/A and ACCOUGH will be investigated to determine a set of descriptive ACCOUGH features as biomarkers of P/A in HNC patients (ACCOUGH-P/A). The FEES examination enables the simultaneous observation of P/A occurrence in HNC patients and the acoustic recording of coughing. Swallowing trials performed during the FEES include 2 × 5 ml of water (IDDSI 0), 2 × 20 ml of water (IDDSI 0), 2 × 5 ml of thickened water (IDDSI 4) and 2 × ¼ of a rusk as solid food (IDDSI 7). All FEES pictures and videos will be recorded for later examination.

##### Swallowing scores

Swallowing scores will be obtained via the Penetration-Aspiration Scale (PAS), the Yale Pharyngeal Residue Severity Rating Scale (YPRS) and the number of swallows. The PAS is used to score the P/A risk after each swallowing trial [54]. The Yale Pharyngeal Residue Severity Rating Scale (YPRS) is a 5-point scale used to rate the severity of residue in the valleculae and pyriform sinuses [55]. YPRS scoring will be carried out before the start of the trials and after each swallowing trial. The number of required dry swallows to clear pooling (pre-swallow secretions and post-swallow food residue) will be counted (< 2, between 2 and 5, > 5), and their efficacy will be verified [13].

##### Voice features

Pre- and post-swallow voice quality will be monitored via acoustic features to investigate whether a change in voice quality occurs with P/A. Fundamental frequency (F0), harmonics-to-noise ratio (HNR), jitter, shimmer and intensity (dB) will be examined before and after each swallow trial to report changes in vocal fold vibration with P/A. Acoustic voice features will be correlated with the acoustic cough features and with P/A swallowing scores.

##### Aerodynamic features

The aerodynamic cough features will include the peak expiratory flow rate (l/s), and the total expired volume (l) reported to be the most reliable aerodynamic predictors of P/A [29, 33, 37]. The aerodynamic cough features will be correlated with the acoustic cough features.

#### Participant timeline {13}

All participants will be assessed once, at a minimum of 3 months following (C)RT.

#### Sample size {14}

It is not possible to predetermine accurately the number of participants because the study is exploratory. Indeed, to our knowledge, no acoustic cough-related features have ever been reported as biomarkers of P/A in the framework of dysphagia. The study therefore starts with healthy subjects to obtain reference data. The minimal number of participants has been fixed based on the empirical rule that the number of participants has to be larger than 30 so that the average feature value approximates the mean feature value irrespective of the underlining distribution, provided that the mean and variance are finite. We have decided on a number of 40 HNC patients with RAD (and, consequently, 40 healthy participants for comparison) based on the available number of oncological follow-up consultations and taking into account refusals or drop-outs.

The preliminary conclusions will be based on the comparison with healthy volunteers of acoustic and aerodynamic features of HNC patients reporting RAD. A pilot group of 10 HNC patients without RAD (CTCAE score = 0) is also included in this study.

#### Recruitment {15}

Eligible healthy participants will be recruited among hospital staff and extra hospital parties. Eligible HNC patients will be recruited by the appointed researchers during the multidisciplinary oncological follow-up consultations. HNC patients will be informed about the study during the consultation and will receive an information letter with contact details. If a participant agrees to participate, the informed consent will be signed prior to the recordings.

The participating centres have a high level of experience in the field of head and neck cancer and dysphagia. They are expected to enable the recruitment of the required number of participants.

### Assignment of interventions: allocation

#### Sequence generation {16a}

Not applicable because this study does not involve randomization.

#### Concealment mechanism {16b}

Not applicable because this study does not involve randomization.

#### Implementation {16c}

Not applicable because this study does not involve randomization.

### Assignment of interventions: blinding

#### Who will be blinded {17a}

Not applicable because this study does not involve blinding.

#### Procedure for unblinding if needed {17b}

Not applicable because this study does not involve blinding.

### Data collection and management

#### Plans for assessment and collection of outcomes {18a}

Data collection will be carried out by one SLP trained with the assessment tools required for this study. All data will be reported in case report forms (CRFs) consistently.

#### Plans to promote participant retention and complete follow-up {18b}

Not applicable because participants are not followed up in the framework of the study.

#### Data management {19}

The datasets generated during the study are not publicly available because they will contain patient data and the informed consent does not foresee sharing data publicly. All participant data will be documented by a research assistant (a certified SLP) and entered in a paper CRF developed for the study. Pseudonymized CRFs will be kept in a locked room at the Jules Bordet Institute, separate from the pseudonymization code sheet that identifies participants. Data will be backed up daily on a password-protected hard disk and in the cloud. The principal investigators and the first author (research assistant) will have access to all the recorded data. Signed informed consents will be securely stored by the study coordinators in a locked room at the Jules Bordet Institute.

#### Confidentiality {27}

All data will be coded and securely stored for 25 years. A sequential identification number will be allocated to each participant registered in the study. This number will identify the participant and must identify samples. To avoid identification errors, the participant’s code (a maximum of 4 alphanumeric characters) and date of birth will be recorded. The responsible investigators will ensure that this study is conducted in agreement with either the Declaration of Helsinki (available on the World Medical Association website (http://www.wma.net)) or the laws and regulations of Belgium, whichever provides the best protection for the participant. Only key-coded data may be reported to the sponsors of the study. The protocol must be approved by the competent ethics committees as required by the applicable national legislation.

#### Plans for collection, laboratory evaluation and storage of biological specimens for genetic or molecular analysis in this trial/future use {33}

Not applicable because this study does not require the collection of biological specimens.

## Statistical methods

### Statistical methods for primary and secondary outcomes {20a}

No data are available in the literature regarding the association of dysphagia with the cough and voice features that will be obtained in this study. Therefore, this study is exploratory, and no a priori hypothesis must be tested. Non-parametrical tests (Wilcoxon and Mann–Whitney *U* tests) will be used for data comparison (acoustic versus aerodynamic data, acoustic data obtained for different samples) with the most recent version of the IBM SPSS Statistics software. For all non-parametrical tests, *p*-values and bootstrapped confidence intervals (95%) for the medians will be reported. Sensibility and specificity of the developed acoustic methods of analysis will also be explored.

The primary conclusions of this exploratory research project will be based on the intention-to-treat principle (ITT). All participants’ data will be analysed except if they withdraw consent of use of their data.

### Interim analyses {21b}

No interim analyses will be carried out because all patients will produce the same tasks, which are considered to be safe.

### Methods for additional analyses (e.g. subgroup analyses) {20b}

A pilot group of 10 HNC patients without RAD (CTCAE score: 0; YPRS score: a; PAS score: 1) is included in this research. Given the high percentage of HNC patients with RAD, we have targeted a smaller group of patients without RAD for this exploratory study.

### Methods in analysis to handle protocol non-adherence and any statistical methods to handle missing data {20c}

Missing data will be assumed to be missing at random (MAR) for all variables. Indeed, this study is exploratory, and unavailable information cannot be interpreted/predicted at this stage. Therefore, multiple imputations with 15 imputations created by predictive models will be conducted, based on the majority of participants with complete data. After the imputations are completed, all of the data (complete and imputed) will be combined, and the analysis will be performed for each imputed-and-completed dataset. Information lacking due to withdrawal or discontinuation of the tasks will not be considered as “missing” and will not be taken into account for the calculation of the missing data.

### Plans to give access to the full protocol, participant-level data and statistical code {31c}

The dataset and identification codes will not be publicly available.

### Oversight and monitoring

#### Composition of the coordinating centre and trial steering committee {5d}

Two principal investigators (a radiation oncologist at Jules Bordet Institute and a SLP at the University of Antwerp) will be designated in each participating centre. The PI will be responsible for identification, recruitment, data collection and completion of informed consent forms, along with follow-up of study patients and adherence to study protocol and investigators brochure. A collaborative agreement between both centres will be signed with regard to the attributed roles and responsibilities.

The trial has three management committees with independent members: the Clinical Projects Committee (CPC), the Review Board Meeting of the Radiation-Oncology Department (RB) and the Thesis Monitoring Committee (TMC). The CPC reviewed and approved the trial from a scientific and a statistical standpoint. The RB will meet regularly (at least every 3 months) to ensure that the trial is progressing according to plan. More regular and individual meetings between the principal investigators and different parts of the research team will be planned as appropriate. The TMC will meet annually to ensure that the PhD is making good progress on schedule.

#### Composition of the data monitoring committee, its role and reporting structure {21a}

A data monitoring committee is not needed because this study is low-risk and not expected to disturb daily clinical practice.

#### Adverse event reporting and harms {22}

An adverse event (AE) is any unfavourable or unintended sign or symptom caused by the trial. Our study is low-risk, and no AE is expected during this trial. Indeed, all the tasks are considered to be safe. Voluntary coughs and voluntary throat clearings will be produced upon request. Cough induction will be carried out via citric acid inhalation, a well-known tussigen with no expected side effects [31]. Natural reflexive coughs due to actual P/A will be recorded during a fiberoptic endoscopic evaluation of swallowing (FEES). The FEES examination will be stopped, when a significant discomfort is experienced by the patient.

Participants will be advised to report immediately any unforeseen adverse event of the trial conduct to the principal investigator.

#### Frequency and plans for auditing trial conduct {23}

The course of the study will be reported annually to the funders.

#### Plans for communicating important protocol amendments to relevant parties (e.g. trial participants, ethical committees) {25}

The protocol amendments will be submitted for approval to the Ethics Committees of the participating centres. All the study staff will receive notice of changes from the study coordinators. The amendment history will be tracked via version and date control of the protocol and associated documents.

#### Dissemination plans {31a}

Participants and clinicians will be informed of the results of this study via peer-reviewed journals and presentations at national and international conferences. A public presentation of the findings will also be planned at the end of the project.

## Discussion

A growing number of studies have demonstrated the reliability of assessing coughing as a clinical marker of dysphagia. One has observed in daily clinical practice that subjective scoring of coughing entails low inter-rater agreement and inconsistencies amongst professional caregivers. Cough airflow-related measures are currently regarded as reliable markers (gold standard) of dysphagia and P/A. However, aerodynamic equipment is not widely available in clinical practice, and it may interfere with an evaluation in a natural setting (during a meal, for instance) because of the presence of a pipe or facemask.

With regard to acoustic analysis, no reliable acoustic cough-related features have been reported yet for assessing RAD in HNC patients, nor for assessing dysphagia and P/A in other populations. The overall goal of our research is to develop methods of acoustic cough analysis with a view to identifying acoustic features as possible markers of swallowing impairments in HNC patients following head and neck cancer treatment. We believe that objective and automatic cough sound analyses may be easily implemented in a natural setting. We aim at developing software running on widely available devices and recording signals with a skin-contact microphone, which does not disturb food intake and does not record external environmental noise.

## Trial status

A tryout has been carried out including 15 healthy participants to develop the first version of the software and for equipment testing (microphones and pre-amplifier) before recruitment. Data collection of the main study started on January 4, 2021, and is planned to end on September 30, 2023. The protocol version used is the V.04 4 March 2020.

## Data Availability

The datasets generated during the current study are not publicly available because they will contain participants’ data and the informed consent does not foresee sharing data publicly. The datasets will be available from the corresponding author upon reasonable request.

## Abbreviations

ACCOUGH: Identification of relevant acoustic cough features in a healthy population
ACCOUCH-P/A: Identification of relevant acoustic cough features as biomarkers of penetration/aspiration in head and neck cancer patients
AE: Adverse event
C2: Dose of tussive agent required to elicit two coughs
CFS: Clinical Frailty Scale
CRF: Case report form
(C)RT: (Chemo)radiotherapy
CTCAE: Common Terminology Criteria for Adverse Events
DCT: Discrete cosine transform
FEES: Fiberoptic endoscopic evaluation of swallowing
HNC patients: Head and neck cancer patients
ITT: Intention-to-treat principle
P/A: Penetration/aspiration
PAS: Penetration-Aspiration Scale
PEFR: Peak expiratory flow rate
RAD: Radiation-associated dysphagia
SLP: Speech-language pathologist
TEV: Total expired volume
YPRS: Yale Pharyngeal Residue Severity Rating Scale
